# Different Responses of Various Chlorophyll Meters to Increasing Nitrogen Supply in Sweet Pepper

**DOI:** 10.3389/fpls.2018.01752

**Published:** 2018-11-27

**Authors:** Francisco M. Padilla, Romina de Souza, M. Teresa Peña-Fleitas, Marisa Gallardo, Carmen Giménez, Rodney B. Thompson

**Affiliations:** ^1^CIAIMBITAL Research Centre for Mediterranean Intensive Agrosystems and Agrifood Biotechnology, Department of Agronomy, University of Almería, Almeria, Spain; ^2^Department of Agronomy, University of Córdoba, Córdoba, Spain

**Keywords:** *Capsicum annuum*, fluorescence, fertilization, optical sensors, transmittance

## Abstract

Intensive vegetable production is commonly associated with excessive nitrogen (N) fertilization and associated environmental problems. Monitoring of crop N status can enhance crop N management. Chlorophyll meters (CMs) could be used to monitor crop N status because leaf chlorophyll (chl) content is strongly related to crop N status. To monitor crop N status, relationships between CM measurements and leaf chl content require evaluation, particularly when excessive N is supplied. The SPAD-502 meter, atLEAF+ sensor, MC-100 Chlorophyll Concentration Meter, and Multiplex sensor were evaluated in sweet pepper with different N supply, throughout the crop, ranging from very deficient to very excessive. CM measurements of all sensors and indices were strongly and positively related to leaf chlorophyll *a* + *b* content with curvilinear relationships over the entire range of chl measured (∼0–80 μg cm^-2^). Measurements with the SPAD-502, and atLEAF+, and of the Multiplex’s simple fluorescence ratio index (SFR) had asymptotic responses to increasing leaf chl. In contrast, the MC-100’s chlorophyll content index (CCI) had a progressively increasing response. At higher chlorophyll *a* + *b* contents (e.g., >40 μg cm^-2^), SPAD-502, atLEAF+ and SFR measurements tended to saturate, which did not occur with CCI. Leaf chl content was most accurately estimated by CCI (*R*^2^ = 0.87), followed by the SPAD-502 meter (*R*^2^ = 0.85). The atLEAF+ sensor was the least accurate (*R*^2^ = 0.76). For leaf chl estimation, CCI measured with the MC-100 meter was the most effective of the four sensors examined because it: (1) most accurately estimated leaf chl content, and (2) had no saturation response at higher leaf chl content. For non-saturating leaf chl content (∼0–40 μg cm^-2^), all indices were sensitive indicators. As excessive applications of N are frequent in intensive vegetable crop production, the capacity of measuring high leaf chl contents without a saturation response is an important consideration for the practical use of chlorophyll meters.

## Introduction

Optimal crop nitrogen (N) management requires that the amount and timing of the N supply be matched to crop demand (Meisinger et al., 2008; Gebbers and Adamchuk, 2010; Ata-Ul-Karim et al., 2016). An effective approach to assist in matching supply to demand is the use of on-farm optical sensors to detect N deficiency, sufficiency or excess (Fox and Walthall, 2008; Samborski et al., 2009; Usha and Singh, 2013; Thompson et al., 2017; Padilla et al., 2018). Optical sensors have several practical characteristics for crop management applications: results are rapidly available, and measurements can be made quickly and periodically throughout a crop (Padilla et al., 2018).

Some optical sensors provide relative measurements of compounds that are sensitive to crop N amount (Fox and Walthall, 2008; Samborski et al., 2009; Tremblay et al., 2012; Padilla et al., 2018). Chlorophyll (chl) is one such N-sensitive compound. Leaf chl content is strongly influenced by leaf N content (Schepers et al., 1996; Cartelat et al., 2005; Samborski et al., 2009) because most leaf N is involved in photosynthesis in several ways (Evans, 1989). Chlorophyll content can be estimated with hand-held chlorophyll meters (CMs) which either clip onto or are positioned close to the leaf surface (Parry et al., 2014; Padilla et al., 2018). All CMs determine the relative chl content per leaf surface area; the measured value is a dimensionless value that strongly relates to the actual amount of chl (Monje and Bugbee, 1992; Markwell et al., 1995; Parry et al., 2014).

Most CMs determine relative leaf chl content by measuring absorbance and transmittance, by the leaf, of (1) red radiation, which chl absorbs, and (2) near infra-red (NIR) radiation, which chl transmits (Fox and Walthall, 2008; Cerovic et al., 2012). Absorbance of red radiation increases with chl resulting in higher CM values (Schepers et al., 1996; Daughtry et al., 2000; Hu et al., 2011). These CMs are often referred to as transmittance-based meters (Padilla et al., 2018). Examples of transmittance-based CMs are the SPAD-502 meter (Konica Minolta, Inc., Tokyo, Japan), the atLEAF+ sensor (FT Green LLC, Wilmington, DE, United States) and the MC-100 Chlorophyll Concentration Meter (Apogee Instruments, Inc., Logan, UT, United States).

Another type of CM estimates chl content from the chlorophyll fluorescence (ChlF) emission ratio of red and far red radiation, emitted from chl, after excitation with radiation ranging from ultra violet (UV) to red (Lichtenthaler et al., 1986; Buschmann, 2007; Tremblay et al., 2012). The ratio of red ChlF to far-red ChlF depends largely on the chl content; because of re-absorption of red ChlF inside the leaf, this ratio decreases with increasing chl content (Buschmann, 2007). These CMs are referred to as fluorimeters or fluorescence-based sensors (Padilla et al., 2018). An example is the Multiplex sensor (Force-A, Orsay, France) (Ben Ghozlen et al., 2010; Tremblay et al., 2012).

The various commercially available CMs differ from one another in the measuring principle (i.e., transmittance *versus* fluorescence), the wavelengths used (Table 1), and the calibration equations used to convert electrical signals into measurement units (Parry et al., 2014; Padilla et al., 2018; Pérez-Patricio et al., 2018). Red radiation absorption of chlorophylls *a* and *b* is nearly equal at approximately 650 nm (Ravinowitch and Govindjee, 1969; Lichtenthaler et al., 1986; Porra et al., 1989); most CMs are designed to measure leaf transmittance at this or similar wavelengths to take into account both types of chlorophyll (Markwell et al., 1995; Uddling et al., 2007; Hunt and Daughtry, 2014). There are slight differences between transmittance-based CMs in the specific red wavelength used (Table 1), which may lead to different sensitivities (Zhu et al., 2012; Taskos et al., 2015).

**Table 1:** Characteristics of leaf chlorophyll meters evaluated in the present study.

Device	Manufacturer	Measuring principle	Wavelengths (nm)	Units
SPAD-502	Minolta (Tokyo, Japan)	Transmittance	650, 940	SPAD units
atLEAF+	FT Green LLC (Wilmington, DE, United States)	Transmittance	660, 940	atLEAF units
MC-100 Chlorophyll Concentration Meter	Apogee Instruments Inc. (Logan, UT, United States)	Transmittance	653, 931	CCI
MULTIPLEX	Force-A (Orsay, France)	Fluorescence	635, 685, 735	SFR_R
MULTIPLEX	Force-A (Orsay, France)	Fluorescence	516, 685, 735	SGR_G


*CCI, Chlorophyll Content Index; SFR_R, Simple Fluorescence Ratio under red excitation; SFR_G, Simple Fluorescence Ratio under green excitation.*

Different behavior of transmittance-based CMs can be expected given the differences in the equations used to calculate the CM measurement value from the ratio of the radiation transmission. The SPAD-502 meter uses a logarithmic equation that includes two proprietary constants, whereas the MC-100 meter uses a simple ratio of the transmission of radiation of NIR and red radiation (Parry et al., 2014). The atLEAF+ sensor uses a logarithmic ratio between NIR and red transmission, similar to that of the SPAD-502 meter (FT Green LLC, personal communication). Overall, the diversity of approaches complicates comparisons between different CMs and, given that most studies use only one CM, there is a requirement for research that provides equations that enable measurements from different CMs to be compared. To date, research has provided conversion equations between the SPAD-502 meter and the MC-100 meter (Parry et al., 2014; Taskos et al., 2015), and between the SPAD-502 meter and atLEAF+ sensor (Zhu et al., 2012), but conversion equations between the MC-100 meter and the atLEAF+ sensor, and between the Multiplex sensor and the SPAD-502 meter, the MC-100 meter and the atLEAF+ sensor, have not been published.

The use of CMs to assess crop N status has been the subject of a large body of research since the 1980s (Padilla et al., 2018). Overall, CM measurements have been shown to be strongly related to leaf and crop N content in a wide range of crops, such as cereals (Arregui et al., 2006; Zhao et al., 2018), tubers (Olivier et al., 2006), vegetables (Wu et al., 2012; Padilla et al., 2015, 2018) and ornamentals (Basyouni et al., 2015; Dunn et al., 2018). Most research with CMs has been conducted with transmittance-based CMs (Fox and Walthall, 2008), but recently, there has been increasing research activity with fluorescence-based CMs (Tremblay et al., 2012; Agati et al., 2013; Padilla et al., 2016). The performance of transmittance-based CMs in comparison to fluorescence-based CMs is unknown.

A number of studies have reported a saturation response of SPAD-502 measurements at higher leaf chl contents (Monje and Bugbee, 1992; Markwell et al., 1995; Cartelat et al., 2005; Uddling et al., 2007), with nearly linear relationship at lower leaf chl contents. With the atLEAF+ sensor, Novichonok et al. (2016) reported appreciable saturation at higher chl contents. Studies with the Multiplex sensor reported linear relationships between chlorophyll indices and leaf chl content, with no indication of saturation of chlorophyll indices (Remorini et al., 2011; Tremblay et al., 2012; Li et al., 2013). However, the range of chl content examined by some of previous studies can be considered as narrow. For example, Tremblay et al. (2012) examined a range of 10–40 μg cm^-2^, whereas leaf chlorophyll content with excessive N supply can be as high as 80 μg cm^-2^ (Monje and Bugbee, 1992; Uddling et al., 2007). Little is known of the performance of various CMs over broad ranges of leaf chl content that include conditions of excessive N supply. As excessive N supply is common in intensive vegetable crop production (Ramos et al., 2002; Ju et al., 2006; Thompson et al., 2007; Soto et al., 2015), the ability of CMs to detect excessive crop N status is an important practical consideration (Lemaire and Gastal, 1997; Thompson et al., 2017; Padilla et al., 2018).

Considering the issues, highlighted above, the objectives of this work were: (1) to assess and compare the nature and strength of the relationships between measurements of different CMs, and leaf chl content over a wide range leaf chl contents, and (2) to develop pair-wise conversion equations between pairs of different CMs. In this work, three transmittance-based meters and a fluorescence-based meter were evaluated in a sweet pepper crop. Sweet pepper was chosen because it is regarded as having a high chl content (Parry et al., 2014). Five treatments of increasing N concentrations in the nutrient solution were applied throughout the crop by fertigation. There were two N deficient treatments, two excessive N treatments and a conventional N management treatment that was regarded as providing a N supply that was close to optimal. The two excessive N treatments enabled assessment of the saturation response of CM measurements to excessive N supply.

## Materials and Methods

### Experimental Site

A sweet pepper (*Capsicum annuum* ‘Melchor’) crop was grown in soil in a plastic greenhouse. The experimental work was conducted at the Experimental Station of the University of Almería (SE Spain, 36° 51′ N, 2° 16′ W and 92 m elevation). The greenhouse has been described elsewhere (Padilla et al., 2014, 2016, 2017); it had polycarbonate walls and a roof of low density polyethylene (LDPE) tri-laminated film (200 μm thickness) with transmittance to photosynthetically active radiation (PAR) of approximately 60%. It had no heating or artificial light, had passive ventilation (lateral side panels and flap roof windows), and an east-west orientation, with crop rows aligned north-south.

The soil was an artificial layered “enarenado” soil (Thompson et al., 2007), consisting of a 30 cm layer of imported silty loam textured soil placed over the original loam soil and a 10 cm layer of fine gravel (mostly 2–5 mm diameter) placed on the imported soil as a mulch (Padilla et al., 2016).

Above-ground drip irrigation was used for combined irrigation and mineral fertilizer application. Drip tape was arranged in paired lines with 0.8 m spacing between lines within each pair, 1.2 m spacing between adjacent pairs of lines, and 0.5 m spacing between drip emitters (3 L h^-1^) within drip lines, giving an emitter density of 2 emitters m^-2^.

The greenhouse was organized into a total of 20 plots, each measuring 6 m × 6 m. There were five N treatments with four replicate plots per treatment, arranged in a randomized block design. Midway between adjacent plots, a 20 cm wide sheet of plastic was vertically positioned from the soil surface to prevent lateral movement of nutrients. Each plot contained three paired lines of drip tape with 12 drip emitters in each line. One plant was positioned 6 cm from and immediately adjacent to each dripper, giving a plant density of 2 plants m^-2^ and 72 plants per replicate plot (Padilla et al., 2016).

### Sweet Pepper Crop

The sweet pepper crop was grown with a summer-winter growing cycle in 2016–2017. The crop was grown from transplanted 5-week-old seedlings, from July 19, 2016 to March 9, 2017 (cropping period of 233 days).

There were five treatments of different N concentrations in the nutrient solution applied by fertigation, that commenced seven days after transplanting (DAT). The treatments were applied in every irrigation throughout the crop. The N treatments were very N deficient (N1), N deficient (N2), conventional N management (N3), excessive N (N4) and very excessive N (N5), according to the N concentration in the applied nutrient solution. The average applied N concentrations in the N1, N2, N3, N4, and N5 treatments during the crop were 2, 5, 10, 13, and 18 mmol N L^-1^, respectively. Complete nutrient solutions were applied to all five treatments to ensure that macro, secondary and micro-nutrients were not limiting. For all treatments, most N was applied as nitrate (NO_3_^-^) (77% of applied N), the rest as ammonium (NH_4_^+^). 600 mm of irrigation was applied during the 6 weeks preceding transplanting to leach residual soil mineral N and salts from the root zone.

Plants were managed following local commercial practice. The crop was physically supported using nylon cords placed vertically and horizontally along the side of the crop. Irrigation was scheduled to maintain soil matric potential (SMP) in the root zone, at 15 cm depth, within -15 to -25 kPa; one tensiometer (Irrometer, Co., Riverside, CA, United States) per plot was used to measure SMP. Temperature was controlled by white-washing the plastic cladding with a CaCO_3_ suspension, 6 days before transplanting (0.75 kg L^-1^) and 36 DAT (0.40 kg L^-1^). The white-washing was removed by natural rainfall, which commenced during the following autumn.

### Chlorophyll Meters

Measurements were made with three leaf-clip sensors, the SPAD-502, the atLEAF+ and the MC-100, and with one proximal sensor, the Multiplex 3.6 (Table 1). Measurements with the SPAD-502, atLEAF+ and MC-100 were made by clipping the sensor onto the leaf, and measurements with the Multiplex 3.6 were made at a distance of 10 cm from the leaf surface, using the 4-cm diameter aperture mask provided with the sensor (Padilla et al., 2016). The measurement areas of each sensor were 6 mm^2^ for the SPAD-502, 13 mm^2^ for the atLEAF+, 63.6 mm^2^ for the MC-100, and 1,257 mm^2^ for the Multiplex.

All three leaf-clip CMs determine the relative content of chl by measuring radiation absorbance in the red and NIR, with some differences between CMs in the wavelengths used. The SPAD-502 measures absorbance at 650 nm (red) and 940 nm (NIR), the atLEAF+ at 660 nm and 940 nm, and the MC-100 at 653 nm and 931 nm. Using the two absorbance values, the meters calculate a dimensionless numerical value which is related to the chl content. The equations employed by SPAD-502 meter and atLEAF+ sensor to calculate the numerical value are confidential. The measurement values are SPAD units, atLEAF units and the CCI, for the SPAD-502 meter, atLEAF+ sensor and MC-100 meter, respectively. CCI is calculated by the MC-100 as the ratio between transmission of radiation at 931 nm divided by transmission of radiation at 653 nm. Values of measurements made with these three meters increase with leaf chl content.

The Multiplex sensor provides a relative measurement (i.e., index) of chl content by generating fluorescence in leaf tissues using multiple light sources (Ben Ghozlen et al., 2010). It makes use of the ChlF emission ratio of red and far red radiation under visible radiation excitation to given an estimation of the chl content (Lichtenthaler et al., 1986; Buschmann, 2007; Tremblay et al., 2012). The Multiplex calculates an index that is directly linked to the chl content, called SFR that is calculated as the ratio between ChlF emission of far-red (FR, 735 nm) divided by ChlF emission of red (R, 685 nm), either under red (635 nm) (SFR_R) or under green (516 nm) (SFR_G) radiation excitation (Ben Ghozlen et al., 2010; Tremblay et al., 2012). It should be noted that the Multiplex calculates SFR indices using the ratio of ChlF of far-red to red, rather the ratio of ChlF of red to far-red (Buschmann, 2007). Consequently, both the SFR_R and SFR_G indices increase with chl content. Measurements with the Multiplex were made with 250 light excitation flashes per individual measurement, under configuration mode #2, using four LED light sources (UV-A, 373 nm; blue, 470 nm; green, 516 nm; and red, 635 nm), ensuring that there was no saturation in each individual channel (Padilla et al., 2016). Detailed descriptions of the Multiplex and its operation are available elsewhere (Ben Ghozlen et al., 2010; Tremblay et al., 2012; Zhang et al., 2012; Agati et al., 2013).

### Chlorophyll Meter Measurements

Chlorophyll meter measurements commenced on September 15, 2016 and were repeated every 2 weeks until December 12, 2016, for a total of six measurement dates which were September 15 (58 DAT), September 29 (72 DAT), October 13 (86 DAT), October 27 (100 DAT), November 22 (126 DAT), and December 12 (146 DAT). The SPAD-502 and MC-100 CMs were zeroed before commencing measurement, on each measurement date. Correct functioning of SPAD-502 meter and Multiplex sensor was verified before commencing measurement, on each measurement date, using a standard plate provided by the manufacturer. Both zeroing and verification of the proper functioning of the sensor were done according to instructions provided with the devices. Measurements were made each day at 7:00 to 9:00 solar time, before the nutrient solutions were applied by fertigation. Individual CM measurements with each sensor were made on six different plants in each of the four replicate plots of the five N treatments; the six plants were located in the central pair of lines of plants in each plot. One measurement per plant was made on the top side of a leaf on the distal part (Yuan et al., 2016). All measurements with each CM were centered on a point midway between the margin and the mid-rib of the leaf (Yuan et al., 2016). The same position on the same leaf was measured with the four CMs in the order: SPAD-502, MC-100, atLEAF+, Multiplex. The time between consecutive measurement with two different sensors was 5–10 s. In total, 24 leaves from different plants were measured, with each CM, in each of the five N treatments for each measurement date.

### Chemical Analysis of Chlorophyll Content

Immediately after measurement with the CMs, each leaf was excised from the plant and a leaf disk (5.6 mm diameter) was taken from a position in which the center of the disk coincided with the measuring point. The disk was obtained using a metal ring; immediately after collection the leaf disk was sealed in a plastic zip-lock bag and frozen. Chlorophyll from leaf disks was extracted with 80% aqueous acetone solvent following Porra et al. (1989) and Padilla et al. (2017). Each disk was ground with 7.5 mL of the solvent in a homogenizer (IKA T25 digital ULTRA-TURRAX, IKA-Werke GmbH & Co. KG, Staufen, Germany). The homogenate was centrifuged at 2,500 rpm for 10 min (NAHITA model 2655, AUXILAB S.L., Beriain, Spain); the supernatant was transferred to another tube and the volume was adjusted to 15 mL by adding the acetone solvent. This extract was diluted using the acetone solvent; the choice of dilution factors of 1:4, 1:8, 1:10, 1:20 or not being diluted, depended on the N treatment (Padilla et al., 2017). Absorbance was then measured at 646.5 nm, 663.5 nm and 750 nm with a spectrophotometer (Zuzi model 4201/20, AUXILAB S.L.) which had been zeroed and auto-calibrated. Dilution was necessary to ensure optimum ranges of absorbance of the spectrophotometer. The concentration of chlorophyll *a*, chlorophyll *b* and chlorophyll *a* + *b* were calculated using the equations described in Porra et al. (1989):

$$Chla=12.25\cdot A^{663.6}-2.55\cdot A^{646.6}$$

$$Chlb=20.31\cdot A^{646.6}-4.91\cdot A^{663.6}$$

$$Chla+b=17.76\cdot A^{646.6}+7.34\cdot A^{663.6}$$

where *A* is the absorbance measurement at the indicated wavelength following subtraction of absorbance at 750 nm, as described by Porra et al. (1989). Absorbance was measured at 646.5 nm and 663.5 nm instead of at 646.6 nm and 663.6, as in the procedure of Porra et al. (1989), because of the resolution of the spectrophotometer (0.5 nm) (Padilla et al., 2017).

### Analysis of Leaf N Content

The remaining half of each sampled leaf was placed in a paper bag and oven dried at 65°C until constant weight. Petioles were discarded. Dry material was ground in a ball mill. The total N content (%) of each sample was determined using a Dumas-type elemental analyzer system (model Rapid N, Elementar, Analysensysteme GmbH, Hanau, Germany).

### Statistics

Data collected on the six dates of measurement were pooled to integrate the widest range of chlorophyll content sampled throughout the sweet pepper crop. Regression analyses were conducted to evaluate the nature and the strength of the relationships, for each CM, between: (1) chl content and CM measurement, and vice versa, (2) leaf N content and chl content, and (3) CM measurements of the various paired CMs (pair-wise comparisons). For the Multiplex sensor, separate regression analyses were conducted for the SFR_R and SFR_G indices. This manuscript will focus on results for the SFR_G as this index performed slightly better than SFR_R; the results of SFR_R are in the Supplementary Material.

Regressions were examined in two ways: (1) with leaf chl content on the *x*-axis, against CM measurements on the *y*-axis, to examine the saturation response of CM measurements with increasing leaf chl contents, and (2) with CMs measurements on the *x*-axis against leaf chl content, on the *y*-axis, to provide equations to estimate chl content from CM measurements. In this sense, the pooling of data collected on the six dates of measurements enabled derivation of relationships that apply for a wide range of chlorophyll content. For consistent use of terminology throughout the manuscript, regressions were classified, according to *R*^2^ values, as very strong (*R*^2^ ≥ 0.85), strong (0.85 > *R*^2^ ≥ 0.7), moderate (0.7 > *R*^2^ ≥ 0.5), weak (0.5 > *R*^2^ ≥ 0.2) and very weak (*R*^2^< 0.2) (Padilla et al., 2016).

In all analyses, linear, quadratic, power, exponential and natural logarithmic regression were considered, and the best was selected using the AIC (Akaike, 1974), which represents the best compromise between highest goodness of fit and smallest degree of regression complexity. The CurveExpert Professional 2.6.0 software (Daniel G. Hyams) was used to compare regressions and to obtain the coefficient of determination (*R*^2^), the SEE, and the equation of the selected regression. The number of data points for each regression, for each CM, was 667–720; each data point represents an individual measurement for a given CM and the corresponding leaf chl content, of each leaf that was measured. Differences in the number of data points were due to connection errors when downloading data from CMs in the field.

To further analyze the saturation response of CM measurement to increasing leaf chl content, separate linear regression analyses were conducted for leaf chlorophyll *a* + *b* content for the ranges 0–40 and of 40–80 μg cm^-2^. The sensitivity of CM measurement to increasing chlorophyll *a* + *b* content was indicated by the differences in the slopes of the linear regressions for these two ranges. For each CM, the slopes of the two regressions for chl content ranges of 0–40 and of 40–80 μg cm^-2^ were compared, for statistically significant differences at *p* < 0.05, using the Comparison of Regression Lines procedure of the Statgraphics Centurion XVII software (StatPoint Technologies Inc., Warrenton, VA, United States). This procedure performs an analysis of variance between the two data sets for the two ranges of chlorophyll content to determine whether there are significant differences between the slopes of the linear regression equations derived from the two data sets. Statistically significant lower slopes for 40–80 μg cm^-2^ compared to 0–40 μg cm^-2^ were considered to indicate reduced sensitivity and to indicate a saturation response.

## Results

### Estimation of Chlorophyll Content From CMs Measurements

The best fit equations to estimate chlorophyll *a* + *b* content (μg cm^-2^) from CM measurements are presented in Table 2; those for the separate estimation of chlorophyll *a* and chlorophyll *b* contents are presented in Supplementary Table S1. The CCI measured with the MC-100 meter provided the most accurate estimation of chlorophyll *a* + *b* content with the highest *R*^2^ value and the lowest SEE value. The next most accurate sensor/index, using these criteria, was the SPAD-502 meter, followed by the SFR index measured with the Multiplex sensor (Table 2). The atLEAF+ sensor provided the least accurate estimation of chlorophyll *a* + *b* content of the sensors/indices examined. There was a tendency for all CMs to estimate chlorophyll *a* content with slightly more accuracy than the chlorophyll *b* content (Supplementary Table S1).

**Table 2:** Equations to estimate chlorophyll *a* + *b* content (μg cm^-2^) from measurements with different chlorophyll meters.

Chlorophyll meter	Equation	Regression	*R*^2^	±SEE	*n*
SPAD-502	Chl = -9.873 + 0.990 × SPAD + 0.0015 × SPAD^2^	Quadratic	0.85	5.97	713
atLEAF+	Chl = 0.078 × atLEAF^1.63^	Power	0.76	7.44	719
MC-100	Chl = 7.953 + 1.026 × CCI – 0.0045 × CCI^2^	Quadratic	0.87	5.65	667
Multiplex	Chl = -8.36 + 29.35 × SFR_R	Linear	0.78	7.14	720
Multiplex	Chl = -6.431 + 27.080 × SFR_G – 1.238 × SFR_G^2^	Quadratic	0.81	6.61	720


*Coefficient of determination (*R*^2^), standard error of the estimate (±SEE) and sample size (*n*) of the regression are shown. CCI is chlorophyll content index, measured with the MC-100 meter; SFR is Simple Fluorescence Ratio, either under red (SFR_R) or green (SFR_G) excitation, measured with the Multiplex sensor.*

### Response of CMs Measurements to Increasing Chlorophyll Content

**FIGURE 1:**
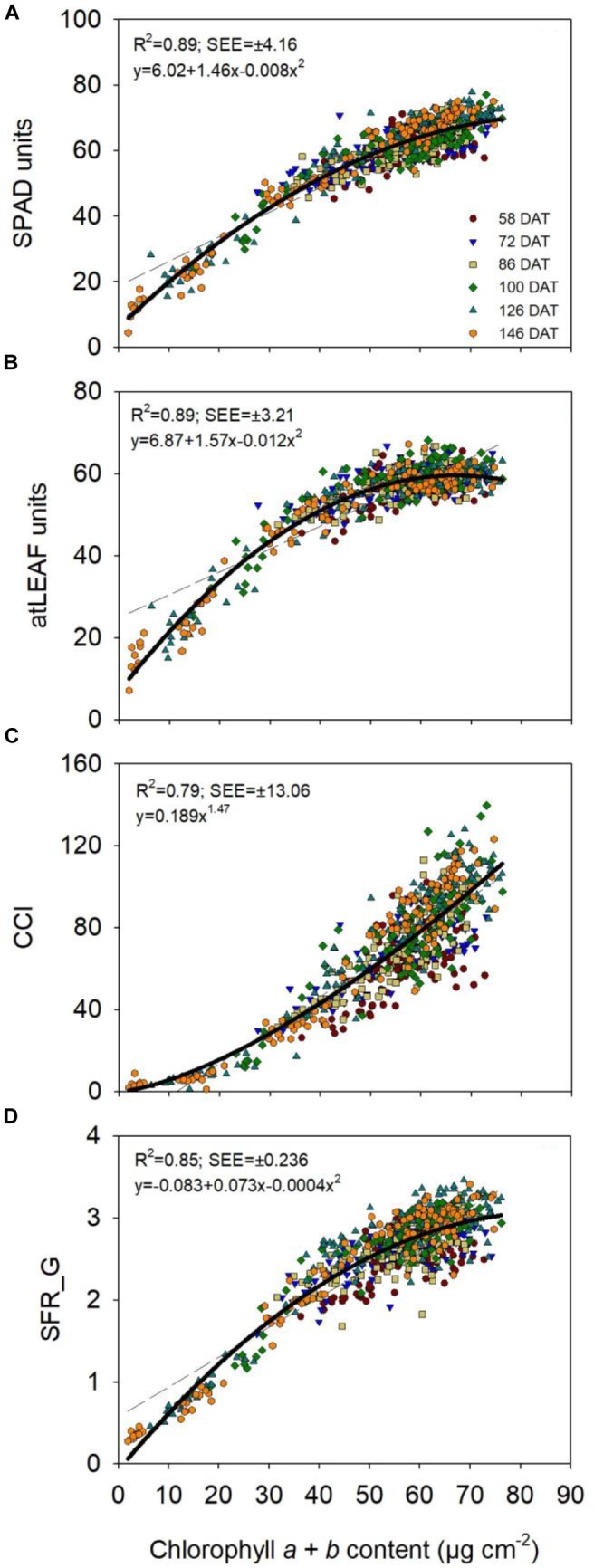
Relationships between chlorophyll *a* + *b* content (μg cm^-2^) and different chlorophyll meter measurements (**A**, SPAD-502 meter; **B**, atLEAF+ sensor; **C**, MC-100 Chlorophyll Concentration Meter; **D**, Multiplex). The coefficient of determination (*R*^2^) and standard error of the estimate (±SEE) values, and the equation of the regression are shown (solid lines). CCI is chlorophyll content index, measured with the MC-100 meter; SFR_G is Simple Fluorescence Ratio under green excitation, measured with the Multiplex sensor. Dotted lines represent the linear regression.

In sweet pepper, chlorophyll *a* + *b* content was positively and strongly related to all CM measurements with curvilinear relationships (Figure 1). The AIC best fit regression was quadratic for the relationships of SPAD units, atLEAF units and SFR_G to leaf chl content, and was a power regression for the relationship of CCI with chl content. Linear regressions also provided a good fit for all sensors/indices (Supplementary Figure S1); however, the curvilinear equations provided a slightly better fit. Comparing quadratic to linear regressions, the respective *R*^2^ values for SPAD-502 were 0.89 vs. 0.85, for atLEAF+ were 0.89 vs. 0.75, and for SFR_G, were 0.85 vs. 0.81. For CCI, the power regression had a slightly better fit than the linear regression, with *R*^2^ values of 0.79 and 0.78, respectively.

**FIGURE 2:**
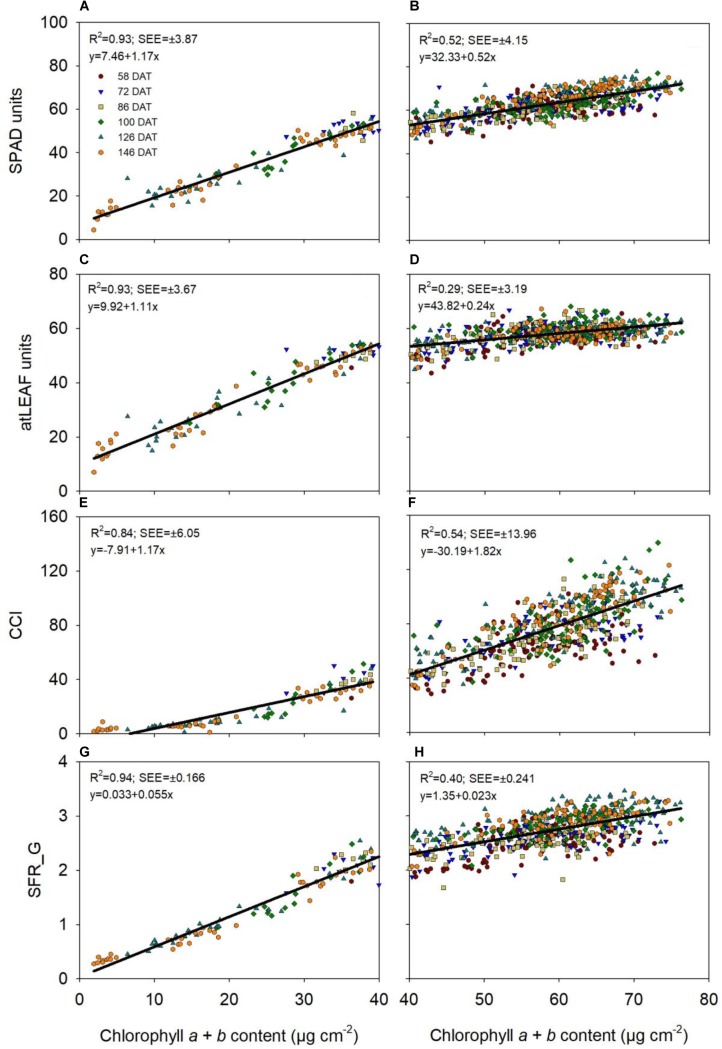
Linear regression between chlorophyll *a* + *b* content, in the range of 0–40 and of 40–80 μg cm^-2^, and different chlorophyll meter measurements (**A,B**, SPAD-502 meter; **C,D**, atLEAF+ sensor; **E,F**, MC-100 Chlorophyll Concentration Meter; **G,H**, Multiplex). The coefficient of determination (*R*^2^) and standard error of the estimate (±SEE) values, and the equation of the regression are shown. CCI is chlorophyll content index, measured with the MC-100 meter; SFR_G is Simple Fluorescence Ratio under green excitation, measured with the Multiplex sensor.

The quadratic regressions showed a tendency of saturation or a plateau response of SPAD-502, atLEAF+ and SFR_G values at higher chlorophyll *a* + *b* contents (Figure 1). SPAD-502, atLEAF+ and SFR_G values increased linearly with increases in chlorophyll *a* + *b* from 0 to approximately 40 μg chl cm^-2^; thereafter, there were proportionately smaller increases in these values for increasing chl levels (Figure 1). For these three sensors, the slopes of the linear regression between CMs measurements and chl content for chlorophyll *a* + *b* content of 40–80 μg cm^-2^ were statistically significantly lower than the slopes of linear regression for chlorophyll *a* + *b* content of 0–40 μg cm^-2^ (*p* < 0.001; Figure 2). The lower slopes in the upper range indicated a loss of sensitivity of the SPAD-502, atLEAF+ and SFR_G values at higher leaf chl content. This effect was strongest with the atLEAF+ sensor, where the slope value for 40–80 μg cm^-2^ was reduced by 78% compared to that for 0–40 μg cm^-2^ (Figure 2). For SPAD-502 and SFR_G values, the slopes for 40–80 μg cm^-2^ were reduced by 56 and 58%, respectively, compared to those for 0–40 μg cm^-2^ (Figure 2).

In contrast, there was no saturation or plateau response of CCI values over the entire range of chlorophyll *a* + *b* levels measured (i.e., ∼0–80 μg cm^-2^, Figure 1). The slopes of linear regression between CCI and chl content for chlorophyll *a* + *b* content of 40–80 μg cm^-2^ were statistically significantly higher (p < 0.001) than the slopes of the equivalent linear regression for 0–40 μg cm^-2^ (*p* < 0.001; Figure 2). The increased slope at the upper range of chl content indicated increased sensitivity of CCI at higher leaf chl contents.

There was notable variability in CCI values when the leaf chl content was 40–80 μg cm^-2^ (Figure 2). At similar leaf chl contents, there was less variability with atLEAF+ and SPAD-502 values (Figure 2).

A similar relationship to that reported for SFR_G was found for SFR_R, in terms of strength of relationships and the saturation response; however, there was a tendency for a slightly weaker relationship to leaf chl for SFR_R than for SFR_G (Supplementary Figures S2, S3).

Similar relationships to those reported for chlorophyll *a* + *b*, were found for chlorophyll *a* and chlorophyll *b* when considered independently (Supplementary Figure S4). There was a tendency for slightly weaker relationships for chlorophyll *b* than for chlorophyll *a* and for combined chlorophyll *a* + *b*, regardless of the CM.

### Relationship Between Chlorophyll Content and Leaf N Content

**FIGURE 3:**
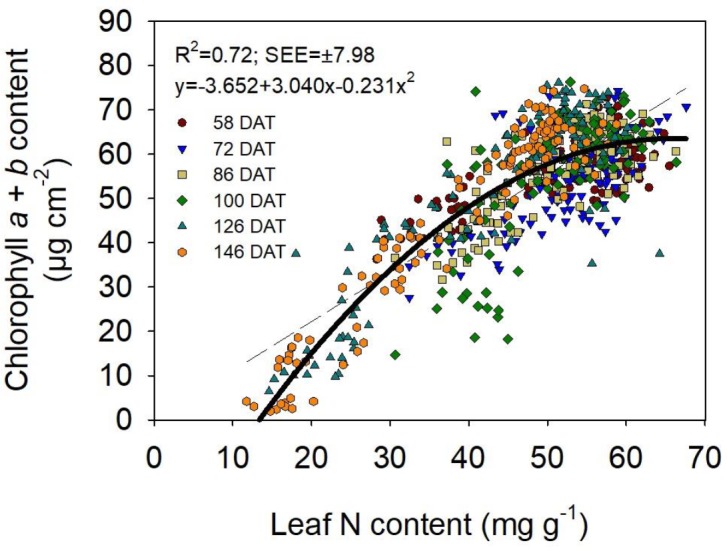
Relationship between leaf N content (mg g^-1^) and chlorophyll *a* + *b* content (μg cm^-2^). The coefficient of determination (*R*^2^) and standard error of the estimate (±SEE) values, and the equation of the quadratic regression are shown (solid line). Dotted line represents the linear regression.

Chlorophyll *a* + *b* content was strongly and positively related to leaf N content (Figure 3). The AIC best-fit regression was the quadratic (*R*^2^ = 0.72) which provided a clearly stronger fit than the linear regression (*R*^2^ = 0.66). There was a tendency for a saturation or a plateau effect of chlorophyll *a* + *b* content at high to very high leaf N contents (∼40–70 mg g^-1^). The relationships between chlorophyll *a* + *b* content and N content, for N contents of approximately 10 mg g^-1^ to 40 mg g^-1^, were close to linear.

A similar quadratic relationship to that obtained for leaf chlorophyll *a* + *b* content versus leaf N content was obtained for the individual relationships for chlorophyll *a* content or chlorophyll *b* content versus leaf N content (Supplementary Figure S5). The relationship for chlorophyll *b* content was weaker than those for chlorophyll *a* and for chlorophyll *a* + *b* contents.

### Relationship Between CMs Measurements

**FIGURE 4:**
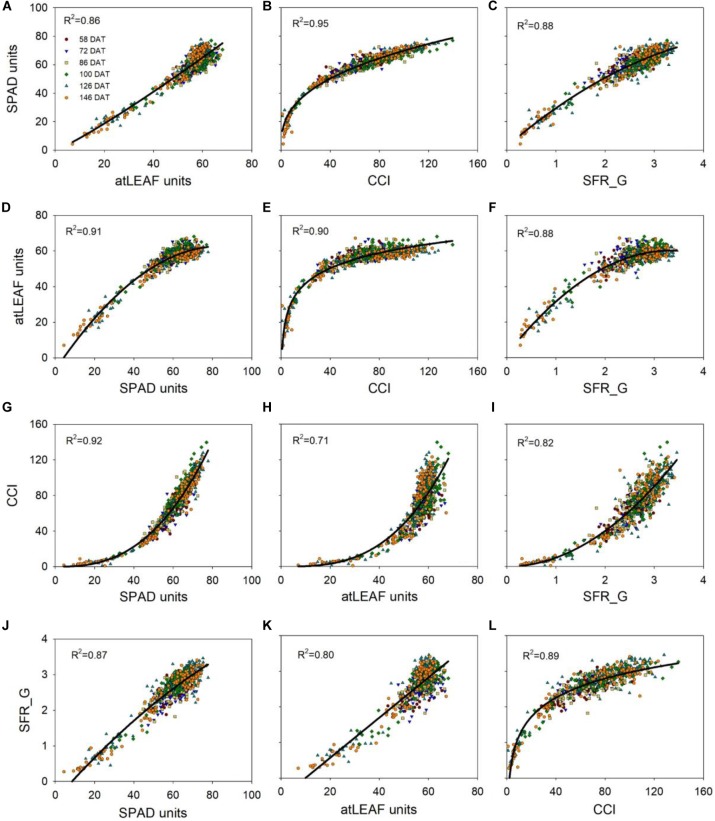
Relationship between measurements of different chlorophyll meters (**A–C**, relationships of SPAD-502 with the rest of CMs; **D–F**, relationships of the atLEAF+ with the rest of CMs; **G–I**, relationships of the MC-100 Chlorophyll Concentration Meter with the rest of CMs; **J–L**, relationships of the Multiplex with the rest of CMs). Coefficient of determination (*R*^2^) values of the regression are shown; the equations and standard errors of the estimate (±SEE) are in Table 3. CCI is chlorophyll content index, measured with the MC-100 meter; SFR_G is the Simple Fluorescence Ratio under green excitation, measured with the Multiplex sensor.

Measurements of the four CMs were either strongly (0.85 > *R*^2^ ≥ 0.7) or very strongly (*R*^2^ ≥ 0.85) related to each other (Figure 4). The strongest relationships were between CCI and SPAD-502 values, and between SPAD-502 and atLEAF+ values. The weakest relationship was between atLEAF+ and CCI values. Regarding the nature of the relationships, near linear relationships with slight curvature occurred for SPAD-502 against atLEAF+, SPAD-502 against SFR_G, SFR_G against SPAD-502, and for SFR_G against atLEAF+. The rest of relationships were more strongly curvilinear. Equations to convert measurements between pairs of CMs are presented in Table 3.

**Table 3:** Equations to convert measurement units of different chlorophyll meters.

Chlorophyll meter	Equation	Regression	*R*^2^	±SEE	*n*
SPAD-502	SPAD = 0.622 × atLEAF^1.136^	Power	0.86	4.65	713
	SPAD = 13.302 × CCI^0.360^	Power	0.95	2.96	660
	SPAD = 2.587 + 29.603 × SFR_G – 2.747 × SFR_G^2^	Quadratic	0.88	4.37	713
atLEAF+	atLEAF = -6.907 + 1.647 × SPAD – 0.010 × SPAD^2^	Quadratic	0.91	2.88	713
	atLEAF = 4.897 + 12.309 × ln(CCI)	Logarithm	0.90	3.22	666
	atLEAF = 1.838 + 34.920 × SFR_G – 5.222 × SFR_G^2^	Quadratic	0.88	3.34	719
MC-100	CCI = 0.001337 × SPAD^2.639^	Power	0.92	8.15	660
	CCI = 0.000405 × atLEAF^2.988^	Power	0.71	15.22	666
	CCI = 9.973 × SFR_G^2.003^	Power	0.82	12.18	667
Multiplex	SFR_G = -0.522 + 0.063 × SPAD – 0.000178 × SPAD^2^	Quadratic	0.87	0.23	713
	SFR_G = -0.560 + 0.056 × atLEAF	Linear	0.80	0.28	719
	SFR_G = -0.602 + 0.775 × ln(CCI)	Logarithm	0.89	0.21	667


*Coefficient of determination (*R*^2^), standard error of the estimate (±SEE) and sample size (*n*) of the regression are shown. CCI is chlorophyll content index, measured with the MC-100 meter; SFR_G is the Simple Fluorescence Ratio under green excitation, measured with the Multiplex sensor.*

Both SFR indices measured with the Multiplex sensor were strongly related to each other in a nearly linear manner (Supplementary Figure S6). Similar relationships to those reported for SFR_G were found for SFR_R, in terms of the nature and strength of relationships with the other four CM measurements (Supplementary Table S2).

## Discussion

Strong (0.85 > *R*^2^ ≥ 0.7) and very strong (*R*^2^ ≥ 0.85) relationships were obtained between measurements of the four CMs (SPAD-502, atLEAF+, MC-100, and Multiplex) and chlorophyll *a* + *b* content, in the range of 0–80 μg cm^-2^, in sweet pepper. Overall, these strong and very strong relationships are consistent with other studies that reported similar relationships in sweet pepper (Madeira et al., 2003) and in other horticultural crops such as tomato (Wu et al., 2012), muskmelon (Azia and Stewart, 2001) and cucumber (Padilla et al., 2017). This confirms that measurements with these CMs can be used as non-destructive indicators of leaf chl content in sweet pepper.

There was a tendency for slightly stronger relationships with chlorophyll *a* than with chlorophyll *b* content, for all CMs. However, this difference was minor given the high *R*^2^ values for the relationships between combined chlorophyll *a* + *b* content and the CM measurements for the CMs examined. Red radiation absorption of chlorophylls *a* and *b* are nearly equal at approximately 650 nm (Ravinowitch and Govindjee, 1969; Lichtenthaler et al., 1986; Porra et al., 1989). Most CMs are designed to measure leaf transmittance at this or similar wavelengths to take into account both types of chlorophyll (Markwell et al., 1995; Uddling et al., 2007; Hunt and Daughtry, 2014).

In this study, the most sensitive CMs for estimating chlorophyll content, considering *R*^2^ and SEE values, were the MC-100 meter and the SPAD-502 sensor; the least sensitive sensor was the atLEAF+. We are unaware of why the sensitivity of the atLEAF+ sensor was lower. It cannot be explained by the wavelengths used for red light transmittance; the value used by the atLEAF+ is 660 nm, which is very closed to the red radiation absorption peak of chlorophyll *a* (Ravinowitch and Govindjee, 1969; Lichtenthaler et al., 1986; Porra et al., 1989), while the values for the MC-100 and SPAD-502 meters of, respectively, 653 and 650 nm, were slightly displaced from this peak. Regardless of the cause, the relatively lower sensitivity of the atLEAF+ sensor reported here is consistent with the scarce relevant literature. Zhu et al. (2012) reported that the SPAD-502 measurements were more strongly related to chl content than atLEAF+ measurements in canola, wheat, barley, potato and corn. Generally similar observations have been reported when comparing the SPAD-502 meter to the MC-100 meter; in grapevine (Taskos et al., 2015), SPAD-502 measurements and CCI were similarly related to chl content.

The results of this study of the SFR indices measured with the Multiplex sensor are not consistent with the results of Li et al. (2013). These authors reported that SFR_R poorly estimated leaf chl content of paddy rice (*R*^2^ = 0.35), and that there was no significant relationship for SFR_G with leaf chl content, which was attributed to the low absorbance of green radiation by chl. In contrast, in the present study, both SFR_G and SFR_R indices were strongly related to chl content and were strongly correlated with one another. Other studies have reported that the two SFR indices, measured with Multiplex, were highly correlated with one another (Quemada et al., 2014; Padilla et al., 2016). The difference between the results of Li et al. (2013) and those of the present and previous studies may be explained by the measurement procedure of Li et al. (2013). Li et al. (2013) measured the whole plant canopy, whereas in the present study and the studies of Quemada et al. (2014) and Padilla et al. (2016) measurements were made on individual leaves at a fixed distance. Canopy measurement with the Multiplex may be affected if there is not full leaf coverage of the measured area, and if there are variations in the distance between the measured surface and the sensor (Ben Ghozlen et al., 2010).

In the present study, measurements with a fluorescence-based sensor were compared with those made with transmittance-based CMs. The results suggest that ChlF-based indices such as SFR_R and SFR_G, measured by the Multiplex, have no clear advantage, in terms of sensitivity or the relationship with leaf chl, compared to transmittance-based measurements made with the MC-100 meter and the SPAD-502 meter. These results suggest the main value of ChlF-based indices may be the combination of these indices with measurement of the relative flavonols content, made with the same sensor, to create combined indices such as the Nitrogen Balance Index (NBI) to estimate crop N status (Tremblay et al., 2012; Agati et al., 2013; Padilla et al., 2014, 2016).

As for the relationship between leaf chlorophyll and N contents, it is widely accepted that leaf chl content is positively related to leaf N content (Evans, 1983; Cartelat et al., 2005); however, the strength and nature of the relationship can differ among C3 plant species (Evans, 1989). In the present study, the relationship between leaf chl and leaf N contents showed both (a) a tendency for saturation of chl content at higher N contents (i.e., approximately from 40 to 70 mg g^-1^), and (b) a nearly linear relationship at lower N contents (i.e., approximately from 10 to 40 mg g^-1^). However, it was possible to establish a linear relationship between leaf chl and N contents over the entire range of leaf N content measured with a slight loss of fit compared to the best-fit quadratic relationship. This suggested that the tendency for saturation of leaf chl content at higher leaf N levels may not be conclusive. Similarly, Evans (1983) reported that chl content increased with increasing leaf N content, in wheat, and that the relationship was slightly curvilinear. In contrast, Cartelat et al. (2005) reported a strong linear relationship between leaf chl and N contents for leaf N contents of 25–50 mg g^-1^, also in wheat. It is not clear if the relationship between leaf chl and N contents is influenced by the range of leaf N contents, possibly changing from linear to non-linear at high to very high leaf N contents.

Regarding the evaluation of saturation of CMs measurements at higher chlorophyll content, in the present study, with sweet pepper, there was a tendency for saturation of SPAD units, atLEAF units and SFR values at higher chl contents (>40 μg cm^-2^). For each of these sensors/indices, there were clear linear relationships at both lower (0–40 μg cm^-2^) and higher (40–80 μg cm^-2^) leaf chl content ranges, but with appreciably smaller slopes and more dispersion in the higher range. The reduction of the slopes for the 40–80 μg cm^-2^ range clearly indicated a loss of sensitivity indicative of saturation at higher leaf chl contents.

For the SPAD-502 meter, the slope of linear regression for the 40–80 μg cm^-2^ chl range was 44% of that for the 0–40 μg cm^-2^ range. A number of studies have reported a saturation response of SPAD-502 measurements at high leaf chl contents (Monje and Bugbee, 1992; Markwell et al., 1995; Cartelat et al., 2005; Uddling et al., 2007), with nearly linear relationship at lower leaf chl contents. The occurrence of, or the lack of, saturation with SPAD-502 measurements reported in some studies (Padilla et al., 2017), maybe related to the range of leaf chl in the leaf material being measured. Leaf chl contents can vary appreciably between species (Parry et al., 2014) and within a species depending on nutrition and plant age (Xiong et al., 2015).

Saturation was very evident with the atLEAF+ sensor, where the slope of linear regression for the 40–80 μg cm^-2^ chl range was only 22% of that for the 0–40 μg cm^-2^ range. Novichonok et al. (2016) also reported a high degree of saturation of atLEAF+ values at high chl contents. The two SFR indices, measured with the Multiplex sensor, also showed strong indications of saturation, with the slope in the 40–80 μg m^-2^ chl range being 42% of that for the 0–40 μg cm^-2^ range. Previous studies with the Multiplex sensor reported linear relationships between SFR indices and leaf chl content, with no indication of saturation of SFR indices (Remorini et al., 2011; Tremblay et al., 2012; Li et al., 2013). It may be that the range of chl content examined by these authors was appreciably narrower than in the present study; for example, Tremblay et al. (2012) examined a range of 10–40 μg cm^-2^.

Unlike the other sensors/indices examined, there was no saturation of CCI, measured with the MC-100 meter, with increasing leaf chl content. The AIC-best fit regression equation was a power relationship, which had a very similar *R*^2^ value to the linear regression for the entire range of leaf chlorophyll *a + b* content examined. The strength of both the power and linear relationships and the similarity of the *R*^2^ values for these two relationships, clearly indicated that CCI values increased proportionally with increasing chl content over the full range of chl content examined (i.e., 0–80 μg cm^-2^). Additionally, when considering the 0–40 and 40–80 μg cm^-2^ chl content ranges separately, the slope for 40–80 μg cm^-2^ was 56% higher than that for 0–40 μg cm^-2^. This indicated increased sensitivity of CCI at higher leaf chl contents. Similar non-saturating responses of CCI were reported by Richardson et al. (2002), Cerovic et al. (2012), and Parry et al. (2014).

It should be mentioned that, in the present work, there was notable variability in CCI values associated with leaf chl contents of 40–80 μg cm^-2^, while there was appreciably less variation with atLEAF+ and SPAD-502 measurements. In this study, the greater sensitivity of the MC-100 meter at high leaf chl contents was associated with lower precision at higher leaf chl contents. However, working with sweet pepper, Parry et al. (2014) did not observe such variation with CCI values. The precision of CCI measurements with the MC-100 meter, at high leaf chl contents, requires further research.

The different saturation behavior of the MC-100 meter compared to the SPAD-502 meter and the atLEAF+ sensor may be explained by differences in the equations used to calculate the CM measurement value from the ratio of the transmission of red radiation and NIR. The SPAD-502 meter uses a logarithmic equation that includes two proprietary constants, whereas CCI, calculated by the MC-100 meter, is a simple ratio of the transmission of radiation of NIR and red radiation (Parry et al., 2014). The atLEAF+ sensor uses a logarithmic ratio between NIR and red transmission, similar to that of the SPAD-502 meter (FT Green LLC, personal communication).

Saturation of CM measurements at higher leaf chl contents is a practical limitation for the use of CMs to assist with crop N management where there is excessive N supply. Considering the overall accuracy for estimating leaf chl content and its sensitivity at high chl contents, CCI measured with the MC-100 meter was the most suitable of the sensors/indices examined for monitoring crop N status in intensive vegetable production. As previously mentioned, future work should examine the variability of CCI values observed at higher leaf chl contents in the present work. Despite the saturation response of the atLEAF+ sensor, this sensor can be seen as a low cost alternative to the MC-100 meter and SPAD-502 meter (Zhu et al., 2012), under non-excessive N conditions, with a retail price nearly ten times lower in the European market.

As for the relationships between CMs measurements, the equations reported in this study, obtained in sweet pepper, to convert SPAD units into CCI units, measured with the MC-100 meter, and vice versa, were very similar to those reported by Parry et al. (2014) on a group of ten species, including pepper, and also by Taskos et al. (2015) with grapevine. The equation to convert SPAD units into CCI in the present study is very similar to that of Parry et al. (2014). There was a slight difference in that Parry et al. (2014) used a three-parameter regression, whereas a two-parameter was used in the present study (Supplementary Figure S7). To convert CCI into SPAD units, there were some minor differences between the equation of the present study and the equations reported by Parry et al. (2014) and Taskos et al. (2015) at low CCI values (i.e., <30), but there was nearly perfect agreement for CCI values >30 (Supplementary Figure S7). Considered together, these observations demonstrate the consistency of the relationship between SPAD units and CCI for a wide range of species and conditions.

For the conversion of SPAD units to atLEAF units, and vice versa, there were large differences between the equations developed in this study, and those reported by Zhu et al. (2012) for a total of five species (canola, wheat, barley, potato and corn) (Supplementary Figure S7). For each of the five species evaluated, the conversion equation suggested by Zhu et al. (2012) was very similar to the global equation calculated for the five species together. Zhu et al. (2012) used linear regression equations while in the present study quadratic and power regressions were obtained. However, fitting linear regressions to the data set of the present study did not result in linear equations that were similar to those reported by Zhu et al. (2012) (data not shown). Compared to most other crop species, sweet pepper has a very high leaf chl content (Parry et al., 2014). The notable lack of sensitivity at high chl contents, reported in the present work, may have contributed to this difference with the results of Zhu et al. (2012).

## Conclusion

The results of the present study suggest that measurements with the SPAD-502 meter, the atLEAF+ sensor, CCI measured with the MC-100 Chlorophyll Concentration Meter, and the Multiplex’s chl indices of SFR_R and SFR_G, can all be used as estimators of leaf chl in sweet pepper. Unlike the SPAD-502 meter, the atLEAF+ sensor, and the two SFR indices measured with the Multiplex, the CCI measured with the MC-100 Chlorophyll Concentration Meter did not have a saturation response in the upper range (40–80 μg cm^-2^) of leaf chl examined. In the upper range of leaf chl contents, there was a high degree of saturation with the atLEAF+ sensor and there was relatively less saturation with the SPAD-502 meter and measurements with the Multiplex’s SFR indices. Considering the accuracy of measurement over the entire range of chl content examined and the relative sensitivity at high chl contents associated with excessive N supply, CCI measured with the MC-100 meter was considered the most suitable of the sensors/indices examined for monitoring crop N status in intensive vegetable production. As excessive applications of N are common in intensive vegetable crop production, the ability of the CCI to measure high leaf chl contents without saturation is an important practical consideration.

## Author Contributions

FP, RT, MG, and TP conceived and designed the experiments. FP, TP, and RdS performed the experiments. FP, TP, and RdS contributed with measurements and sample analysis. CG contributed with laboratory resources and analysis. FP analyzed the data. RT provided ideas for data analysis. FP and RT wrote the paper. MG and CG contributed to the editing.

## Conflict of Interest Statement

The authors declare that the research was conducted in the absence of any commercial or financial relationships that could be construed as a potential conflict of interest.

## Supplementary Material

Figure S1 Linear relationship between chlorophyll *a* + *b* content (μg cm^-2^) and different chlorophyll meter measurements. The coefficient of determination (R^2^) and standard error of the estimate ( ± SEE) values, and the equation of the regression are shown (solid lines). CCI is chlorophyll content index, measured with the MC-100 meter; SFR_G is Simple Fluorescence Ratio under green excitation, measured with the Multiplex sensor.

Figure S2 Relationship between chlorophyll *a* + *b* content (μg cm^-2^) and the Simple Fluorescence Ratio under red excitation (SFR_R), measured with the Multiplex sensor. Coefficient of determination (R^2^), standard error of the estimate ( ± SEE) and equation of the regression are shown (solid line). The dotted line represents the linear regression.

Figure S3 Liner regression between chlorophyll *a* + *b* content, in the range of 0 to 40 and of 40 to 80 μg cm^-2^, and the Simple Fluorescence Ratio under red excitation (SFR_R), measured with the Multiplex sensor. Coefficient of determination (R^2^), standard error of the estimate ( ± SEE) and equation are shown.

Figure S4 Relationships between chlorophyll *a* and chlorophyll *b* contents (μg cm^-2^) and different chlorophyll meter measurements. Coefficient of determination (R^2^), standard error of the estimate ( ± SEE) and equation of the regression are shown (solid lines). CCI is chlorophyll content index, measured with the MC-100 meter; SFR is Simple Fluorescence Ratio, either under red (SFR_R) or green (SFR_G) excitation, measured with the Multiplex sensor. Dotted lines represent the linear regression.

Figure S5 Relationship between leaf N content (mg g^-1^) and chlorophyll *a* and chlorophyll *b* contents (μg cm^-2^). Coefficient of determination (R^2^), standard error of the estimate ( ± SEE) and equation of the regression are shown (solid lines). Dotted lines represent the linear regression.

Figure S6 Relationship between measurements of different chlorophyll meters with the Simple Fluorescence Ratio under red excitation (SFR_R), measured with the Multiplex sensor. Coefficient of determination (R^2^) of the regression is shown; equations and standard errors of the estimate ( ± SEE) are in Supplementary Table S2. CCI is chlorophyll content index, measured with the MC-100 meter.

Figure S7 Comparison of relationships between: SPAD units and CCI values (a), CCI values and SPAD units (b), SPAD units and atLEAF units (c), and atLEAF units and SPAD units (d), of the present study and the literature. CCI is chlorophyll content index, measured with the MC-100 meter.

Table S1 Equations to estimate chlorophyll *a* and chlorophyll *b* contents (μg cm^-2^) from measurements with different chlorophyll meters. Coefficient of determination (R^2^), standard error of the estimate ( ± SEE) and sample size (*n*) of the regression are shown. CCI is chlorophyll content index, measured with the MC-100 meter; SFR is Simple Fluorescence Ratio, either under red (SFR_R) or green (SFR_G) excitation, measured with the Multiplex sensor.

Table S2 Equations to convert measurement units of different chlorophyll meters with the Simple Fluorescence Ratio under red excitation (SFR_R), measured with the Multiplex sensor. Coefficient of determination (R^2^), standard error of the estimate ( ± SEE) and sample size (*n*) of the regression are shown. CCI is chlorophyll content index, measured with the MC-100 meter; SFR_G is the Simple Fluorescence Ratio under green excitation.

## Abbreviations

AIC: Akaike Information Criterion
CCI: chlorophyll content index
chl: chlorophyll
ChlF: chlorophyll fluorescence
CM: chlorophyll meter
DAT: days after transplanting
N: nitrogen
*R*^2^: coefficient of determination
SEE: standard error of the estimate
SFR: simple fluorescence ratio

## References

[B1] Agati G. Foschi L. Grossi N. Guglielminetti L. Cerovic Z. G. Volterrani M. (2013). Fluorescence-based versus reflectance proximal sensing of nitrogen content in *Paspalum vaginatum* and *Zoysia matrella* turfgrasses. *Eur. J. Agron.* 45 39–51. 10.1016/j.eja.2012.10.011

[B2] Akaike H. (1974). New look at the statistical model identification. *IEEE Trans. Autom. Control* 19 716–723. 10.1109/TAC.1974.1100705

[B3] Arregui L. M. Lasa B. Lafarga A. Irañeta I. Baroja E. Quemada M. (2006). Evaluation of chlorophyll meters as tools for N fertilization in winter wheat under humid Mediterranean conditions. *Eur. J. Agron.* 24 140–148. 10.1016/j.eja.2005.05.005

[B4] Ata-Ul-Karim S. T. Cao Q. Zhu Y. Tang L. Rehmani M. I. A. Cao W. (2016). Non-destructive assessment of plant nitrogen parameters using leaf chlorophyll measurements in rice. *Front. Plant Sci.* 7:1829. 10.3389/fpls.2016.01829 28018373 PMC5156736

[B5] Azia F. Stewart K. A. (2001). Relationships between extractable chlorophyll and SPAD values in muskmelon leaves. *J. Plant Nutr.* 24 961–966. 10.1081/PLN-100103784

[B6] Basyouni R. Dunn B. L. Goad C. (2015). Use of nondestructive sensors to assess nitrogen status in potted poinsettia (*Euphorbia pulcherrima* L. (Willd. ex Klotzsch)) production. *Sci. Hortic.* 192 47–53. 10.1016/j.scienta.2015.05.011

[B7] Ben Ghozlen N. Cerovic Z. G. Germain C. Toutain S. Latouche G. (2010). Non-destructive optical monitoring of grape maturation by proximal sensing. *Sensors* 10 10040–10068. 10.3390/s101110040 22163456 PMC3231004

[B8] Buschmann C. (2007). Variability and application of the chlorophyll fluorescence emission ratio red/far-red of leaves. *Photosynth. Res.* 92 261–271. 10.1007/s11120-007-9187-8 17525834

[B9] Cartelat A. Cerovic Z. G. Goulas Y. Meyer S. Lelarge C. Prioul J. L. (2005). Optically assessed contents of leaf polyphenolics and chlorophyll as indicators of nitrogen deficiency in wheat (*Triticum aestivum* L.). *Field Crops Res.* 91 35–49. 10.1016/j.fcr.2004.05.002

[B10] Cerovic Z. G. Masdoumier G. Ghozlen N. B. Latouche G. (2012). A new optical leaf-clip meter for simultaneous non-destructive assessment of leaf chlorophyll and epidermal flavonoids. *Physiol. Plant.* 146 251–260. 10.1111/j.1399-3054.2012.01639.x 22568678 PMC3666089

[B11] Daughtry C. S. T. Walthall C. L. Kim M. S. de Colstoun E. B. McMurtrey J. E. (2000). Estimating corn leaf chlorophyll concentration from leaf and canopy reflectance. *Remote Sens. Environ.* 74 229–239. 10.1016/S0034-4257(00)00113-9

[B12] Dunn B. L. Singh H. Goad C. (2018). Relationship between chlorophyll meter readings and nitrogen in poinsettia leaves. *J. Plant Nutr.* 41 1566–1575. 10.1080/01904167.2018.1459697

[B13] Evans J. R. (1983). Nitrogen and photosynthesis in the flag leaf of wheat (Triticum aestivum L.). *Plant Physiol.* 72 297–302. 10.1104/pp.72.2.297 16662996 PMC1066227

[B14] Evans J. R. (1989). Photosynthesis and nitrogen relationships in leaves of C3 plants. *Oecologia* 78 9–19. 10.1007/BF00377192 28311896

[B15] Fox R. H. Walthall C. L. (2008). “Crop monitoring technologies to assess nitrogen status,” in *Nitrogen in Agricultural Systems, Agronomy Monograph No. 49*, eds Schepers J. S. Raun W. R. (Madison, WI: American Society of Agronomy, Crop Science Society of America, Soil Science Society of America), 647–674.

[B16] Gebbers R. Adamchuk V. I. (2010). Precision agriculture and food security. *Science* 327 828–831. 10.1126/science.1183899 20150492

[B17] Hu J. He D. Yang P. (2011). Study on plant nutrition indicator using leaf spectral transmittance for nitrogen detection. *Adv. Inf. Commun. Technol.* 347 504–513. 10.1007/978-3-642-18369-0_60

[B18] Hunt E. R. Jr. Daughtry C. S. T. (2014). Chlorophyll meter calibrations for chlorophyll content using measured and simulated leaf transmittances. *Agron. J.* 106 931–939. 10.2134/agronj13.0322

[B19] Ju X. T. Kou C. L. Zhang F. S. Christie P. (2006). Nitrogen balance and groundwater nitrate contamination: comparison among three intensive cropping systems on the North China plain. *Environ. Pollut.* 143 117–125. 10.1016/j.envpol.2005.11.005 16364521

[B20] Lemaire G. Gastal F. (1997). “Nitrogen uptake and distribution in plant canopies,” in *Diagnosis of the Nitrogen Status in Crop*, ed. Lemaire G. (Heidelberg: Springer-Verlag), 3–43. 10.1007/978-3-642-60684-7_1

[B21] Li J. W. Zhang J. X. Zhao Z. Lei X. D. Xu X. L. Lu X. X. (2013). Use of fluorescence-based sensors to determine the nitrogen status of paddy rice. *J. Agric. Sci.* 151 862–871. 10.1017/S0021859612001025

[B22] Lichtenthaler H. K. Buschmann C. Rinderle U. Schmuck G. (1986). Application of chlorophyll fluorescence in ecophysiology. *Radiat. Environ. Biophys.* 25 297–308. 10.1007/BF01214643 3823375

[B23] Madeira A. C. Ferreira A. De Varennes A. Vieira M. I. (2003). SPAD meter versus tristimulus colorimeter to estimate chlorophyll content and leaf color in sweet pepper. *Commun. Soil Sci. Plant Anal.* 34 2461–2470. 10.1081/CSS-120024779

[B24] Markwell J. Osterman J. C. Mitchell J. L. (1995). Calibration of the Minolta SPAD-502 leaf chlorophyll meter. *Photosynth. Res.* 46 467–472. 10.1007/BF00032301 24301641

[B25] Meisinger J. J. Schepers J. S. Raun W. R. (2008). “Crop nitrogen requirement and fertilization,” in *Nitrogen in Agricultural Systems, Agronomy Monograph No. 49*, eds Schepers J. S. Raun W. R. (Madison, WI: American Society of Agronomy, Crop Science Society of America, Soil Science Society of America), 563–612.

[B26] Monje O. A. Bugbee B. (1992). Inherent limitations of nondestructive chlorophyll meters: a comparison of two types of meters. *HortScience* 27 69–71. 11537728

[B27] Novichonok E. V. Novichonok A. O. Kurbatova J. A. Markovskaya E. F. (2016). Use of the atLEAF+ chlorophyll meter for a nondestructive estimate of chlorophyll content. *Photosynthetica* 54 130–137. 10.1007/s11099-015-0172-8

[B28] Olivier M. Goffart J. P. Ledent J. F. (2006). Threshold value for chlorophyll meter as decision tool for nitrogen management of potato. *Agron. J.* 98 496–506. 10.2134/agronj2005.0108

[B29] Padilla F. M. Gallardo M. Peña-Fleitas M. T. de Souza R. Thompson R. B. (2018). Proximal optical sensors for nitrogen management of vegetable crops: a review. *Sensors* 18:E2083. 10.3390/s18072083 29958482 PMC6069161

[B30] Padilla F. M. Peña-Fleitas M. T. Gallardo M. Giménez C. Thompson R. B. (2017). Derivation of sufficiency values of a chlorophyll meter to estimate cucumber nitrogen status and yield. *Comput. Electron. Agric.* 141 54–64. 10.1016/j.compag.2017.07.005

[B31] Padilla F. M. Peña-Fleitas M. T. Gallardo M. Thompson R. B. (2015). Threshold values of canopy reflectance indices and chlorophyll meter readings for optimal nitrogen nutrition of tomato. *Ann. Appl. Biol.* 166 271–285. 10.1111/aab.12181

[B32] Padilla F. M. Peña-Fleitas M. T. Gallardo M. Thompson R. B. (2016). Proximal optical sensing of cucumber crop N status using chlorophyll fluorescence indices. *Eur. J. Agron.* 73 83–97. 10.1016/j.eja.2015.11.001

[B33] Padilla F. M. Teresa Peña-Fleitas M. Gallardo M. Thompson R. B. (2014). Evaluation of optical sensor measurements of canopy reflectance and of leaf flavonols and chlorophyll contents to assess crop nitrogen status of muskmelon. *Eur. J. Agron.* 58 39–52. 10.1016/j.eja.2014.04.006

[B34] Parry C. Blonquist J. M. Bugbee B. (2014). *In situ* measurement of leaf chlorophyll concentration: analysis of the optical/absolute relationship. *Plant Cell Environ.* 37 2508–2520. 10.1111/pce.12324 24635697

[B35] Pérez-Patricio M. Camas-Anzueto L. J. Sanchez-Alegría A. Aguilar-González A. Gutiérrez-Miceli F. Escobar-Gómez E. (2018). Optical method for estimating the chlorophyll contents in plant leaves. *Sensors* 18:E650. 10.3390/s18020650 29470432 PMC5855050

[B36] Porra R. J. Thompson W. A. Kriedemann P. E. (1989). Determination of accurate extinction coefficients and simultaneous equations for assaying chlorophylls a and b extracted with four different solvents: verification of the concentration of chlorophyll standards by atomic absorption spectroscopy. *BBA Bioenerg.* 975 384–394. 10.1016/S0005-2728(89)80347-0

[B37] Quemada M. Gabriel J. Zarco-Tejada P. (2014). Airborne hyperspectral images and ground-level optical sensors as assessment tools for maize nitrogen fertilization. *Remote Sens.* 6 2940–2962. 10.3390/rs6042940

[B38] Ramos C. Agut A. Lidon A. L. (2002). Nitrate leaching in important horticultural crops of the Valencian Community region (Spain). *Environ. Pollut.* 118 215–223. 10.1016/S0269-7491(01)00314-1 11939284

[B39] Ravinowitch E. Govindjee (1969). Photosynthesis. New York, NY: John Wiley & Sons, Inc.

[B40] Remorini D. Tardelli F. Massai R. Guidi L. Degl’Innocenti E. Agati G. (2011). A non-destructive fluorescence method applied to the assessment of the quality of kiwifruit. *Acta Hortic.* 913 547–552. 10.17660/ActaHortic.2011.913.74

[B41] Richardson A. D. Duigan S. P. Berlyn G. P. (2002). An evaluation of noninvasive methods to estimate foliar chlorophyll content. *New Phytol.* 153 185–194. 10.1046/j.0028-646X.2001.00289.x

[B42] Samborski S. M. Tremblay N. Fallon E. (2009). Strategies to make use of plant sensors-based diagnostic information for nitrogen recommendations. *Agron. J.* 101 800–816. 10.2134/agronj2008.0162Rx

[B43] Schepers J. S. Blackmer T. M. Wilhelm W. W. Resende M. (1996). Transmittance and reflectance measurements of corn leaves from plants with different nitrogen and water supply. *J. Plant Physiol.* 148 523–529. 10.1016/S0176-1617(96)80071-X

[B44] Soto F. Gallardo M. Thompson R. B. Peña-Fleitas M. T. Padilla F. M. (2015). Consideration of total available N supply reduces N fertilizer requirement and potential for nitrate leaching loss in tomato production. *Agric. Ecosyst. Environ.* 200 62–70. 10.1016/j.agee.2014.10.022

[B45] Taskos D. G. Koundouras S. Stamatiadis S. Zioziou E. Nikolaou N. Karakioulakis K. (2015). Using active canopy sensors and chlorophyll meters to estimate grapevine nitrogen status and productivity. *Precis. Agric.* 16 77–98. 10.1007/s11119-014-9363-8

[B46] Thompson R. B. Martinez-Gaitan C. Gallardo M. Gimenez C. Fernandez M. D. (2007). Identification of irrigation and N management practices that contribute to nitrate leaching loss from an intensive vegetable production system by use of a comprehensive survey. *Agric. Water Manag.* 89 261–274. 10.1016/j.agwat.2007.01.013

[B47] Thompson R. B. Tremblay N. Fink M. Gallardo M. Padilla F. M. (2017). “Tools and strategies for sustainable nitrogen fertilisation of vegetable crops,” in *Advances in Research on Fertilization Management in Vegetable Crops*, eds Tei F. Nicola S. Benincasa P. (Heidelberg: Springer), 11–63. 10.1007/978-3-319-53626-2_2

[B48] Tremblay N. Wang Z. Cerovic Z. G. (2012). Sensing crop nitrogen status with fluorescence indicators. A review. *Agron. Sustain. Dev.* 32 451–464. 10.1007/s13593-011-0041-1

[B49] Uddling J. Gelang-Alfredsson J. Piikki K. Pleijel H. (2007). Evaluating the relationship between leaf chlorophyll concentration and SPAD-502 chlorophyll meter readings. *Photosynth. Res.* 91 37–46. 10.1007/s11120-006-9077-5 17342446

[B50] Usha K. Singh B. (2013). Potential applications of remote sensing in horticulture—A review. *Sci. Hortic.* 153 71–83. 10.1016/j.scienta.2013.01.008

[B51] Wu X. Guo J. Zhao C. Chen L. Zhang Y. Fang Z. (2012). Research and application of non-destructive testing diagnosis technology of tomato. *Sens. Lett.* 10 666–669. 10.1166/sl.2012.1889

[B52] Xiong D. Chen J. Yu T. Gao W. Ling X. Li Y. (2015). SPAD-based leaf nitrogen estimation is impacted by environmental factors and crop leaf characteristics. *Sci. Rep.* 5:13389. 10.1038/srep13389 26303807 PMC4548214

[B53] Yuan Z. Cao Q. Zhang K. Ata-Ul-Karim S. T. Tian Y. Zhu Y. (2016). Optimal leaf positions for SPAD meter measurement in rice. *Front. Plant Sci.* 7:719. 10.3389/fpls.2016.00719 27303416 PMC4880590

[B54] Zhang Y. Tremblay N. Zhu J. (2012). A first comparison of Multiplex^®^ for the assessment of corn nitrogen status. *J. Food Agric. Environ.* 10 1008–1016.

[B55] Zhao B. Ata-Ul-Karim S. T. Liu Z. Zhang J. Xiao J. Liu Z. (2018). Simple assessment of nitrogen nutrition index in summer maize by using chlorophyll meter readings. *Front. Plant Sci.* 9:11. 10.3389/fpls.2018.00011 29403521 PMC5780453

[B56] Zhu J. Tremblay N. Liang Y. (2012). Comparing SPAD and atLEAF values for chlorophyll assessment in crop species. *Can. J. Soil Sci.* 92 645–648. 10.4141/cjss2011-100

