# Supplementary material for: Different Responses of Various Chlorophyll Meters to Increasing Nitrogen Supply in Sweet Pepper
Source: Front Plant Sci. 2018 Nov 27;9:1752. doi: 10.3389/fpls.2018.01752 (PMC6277906; doi:10.3389/fpls.2018.01752)
Supplement: Figure S1 — Linear relationship between chlorophyll a + b content (μg cm-2) and different chlorophyll meter measurements. The coefficient of determination (R2) and standard error of the estimate ( ± SEE) values, and the equation of the regression are shown (solid lines). CCI is chlorophyll content index, measured with the MC-100 meter; SFR_G is Simple Fluorescence Ratio under green excitation, measured with the Multiplex sensor. [file Image_1.pdf]

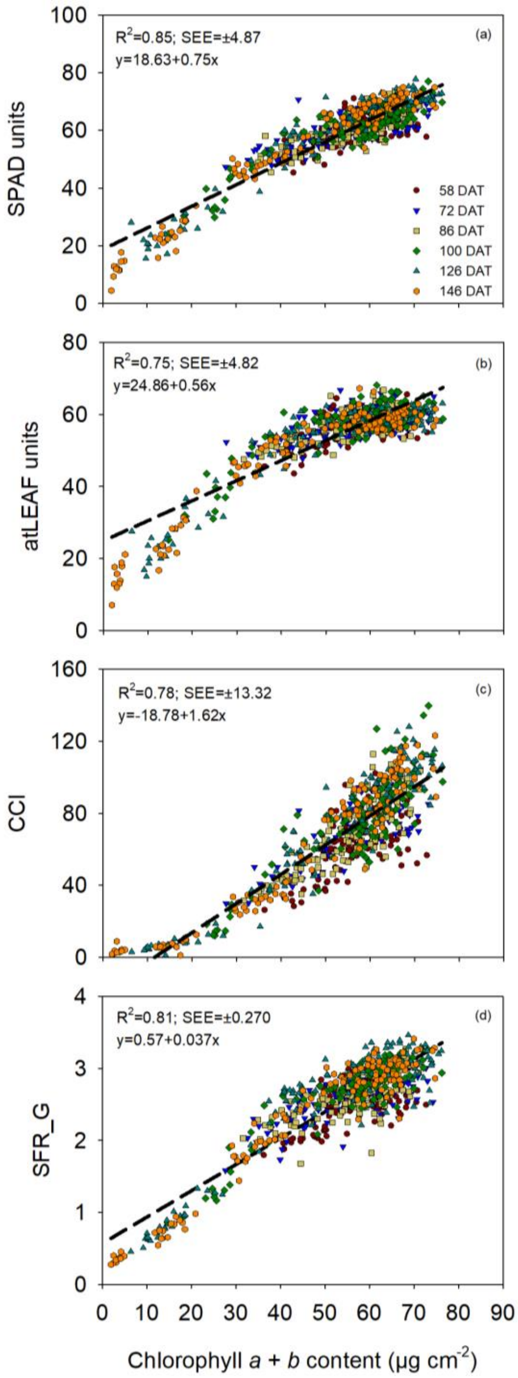

Figure S1. Linear relationship between chlorophyll *a* + *b* content (µg cm^{-2}) and different chlorophyll meter measurements. The coefficient of determination (R^2) and standard error of the estimate (±SEE) values, and the equation of the regression are shown (solid lines). CCI is chlorophyll content index, measured with the MC-100 meter; SFR_G is Simple Fluorescence Ratio under Green excitation, measured with the Multiplex sensor.
