# Supplementary material for: Different Responses of Various Chlorophyll Meters to Increasing Nitrogen Supply in Sweet Pepper
Source: Front Plant Sci. 2018 Nov 27;9:1752. doi: 10.3389/fpls.2018.01752 (PMC6277906; doi:10.3389/fpls.2018.01752)
Supplement: Figure S2 — Relationship between chlorophyll a + b content (μg cm-2) and the Simple Fluorescence Ratio under red excitation (SFR_R), measured with the Multiplex sensor. Coefficient of determination (R2), standard error of the estimate ( ± SEE) and equation of the regression are shown (solid line). The dotted line represents the linear regression. [file Image_2.pdf]

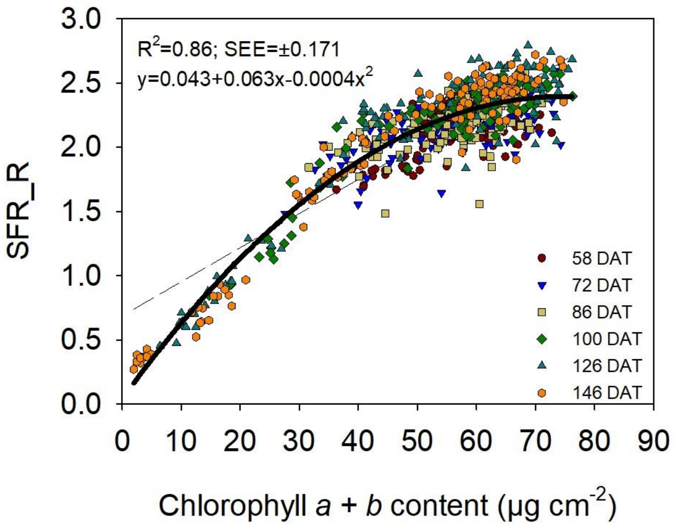

Figure S2. Relationship between chlorophyll *a* + *b* content (µg cm^{-2}) and the Simple Fluorescence Ratio under red excitation (SFR_R), measured with the Multiplex sensor. Coefficient of determination (R^2), standard error of the estimate (±SEE) and equation of the regression are shown (solid line). The dotted line represents the linear regression.
