# Supplementary material for: Different Responses of Various Chlorophyll Meters to Increasing Nitrogen Supply in Sweet Pepper
Source: Front Plant Sci. 2018 Nov 27;9:1752. doi: 10.3389/fpls.2018.01752 (PMC6277906; doi:10.3389/fpls.2018.01752)
Supplement: Figure S3 — Liner regression between chlorophyll a + b content, in the range of 0 to 40 and of 40 to 80 μg cm-2, and the Simple Fluorescence Ratio under red excitation (SFR_R), measured with the Multiplex sensor. Coefficient of determination (R2), standard error of the estimate ( ± SEE) and equation are shown. [file Image_3.pdf]

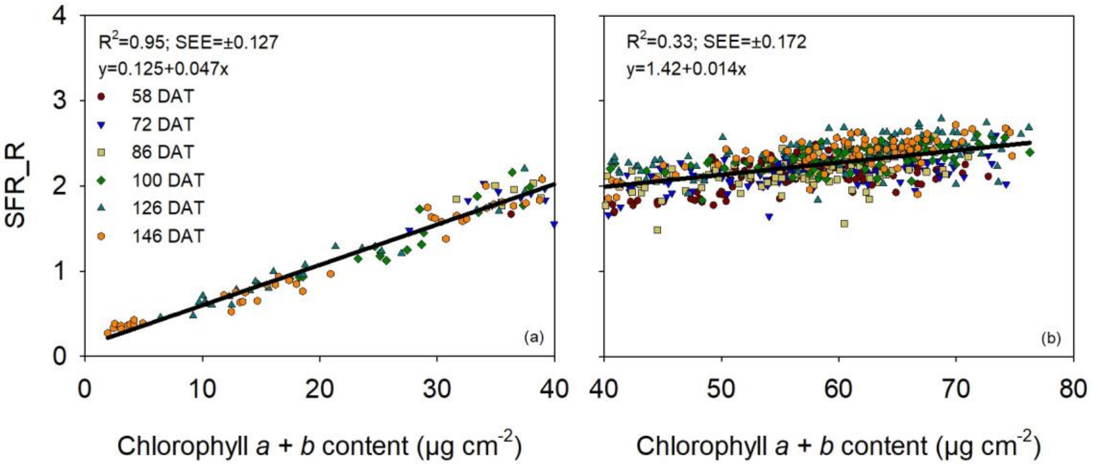

Figure S3. Liner regression between chlorophyll *a* + *b* content, in the range of 0 to 40 and of 40 to 80 µg cm^{-2}, and the Simple Fluorescence Ratio under red excitation (SFR_R), measured with the Multiplex sensor. Coefficient of determination (R^2), standard error of the estimate (±SEE) and equation are shown.
