# Supplementary material for: Different Responses of Various Chlorophyll Meters to Increasing Nitrogen Supply in Sweet Pepper
Source: Front Plant Sci. 2018 Nov 27;9:1752. doi: 10.3389/fpls.2018.01752 (PMC6277906; doi:10.3389/fpls.2018.01752)
Supplement: Figure S4 — Relationships between chlorophyll a and chlorophyll b contents (μg cm-2) and different chlorophyll meter measurements. Coefficient of determination (R2), standard error of the estimate ( ± SEE) and equation of the regression are shown (solid lines). CCI is chlorophyll content index, measured with the MC-100 meter; SFR is Simple Fluorescence Ratio, either under red (SFR_R) or green (SFR_G) excitation, measured with the Multiplex sensor. Dotted lines represent the linear regression. [file Image_4.pdf]

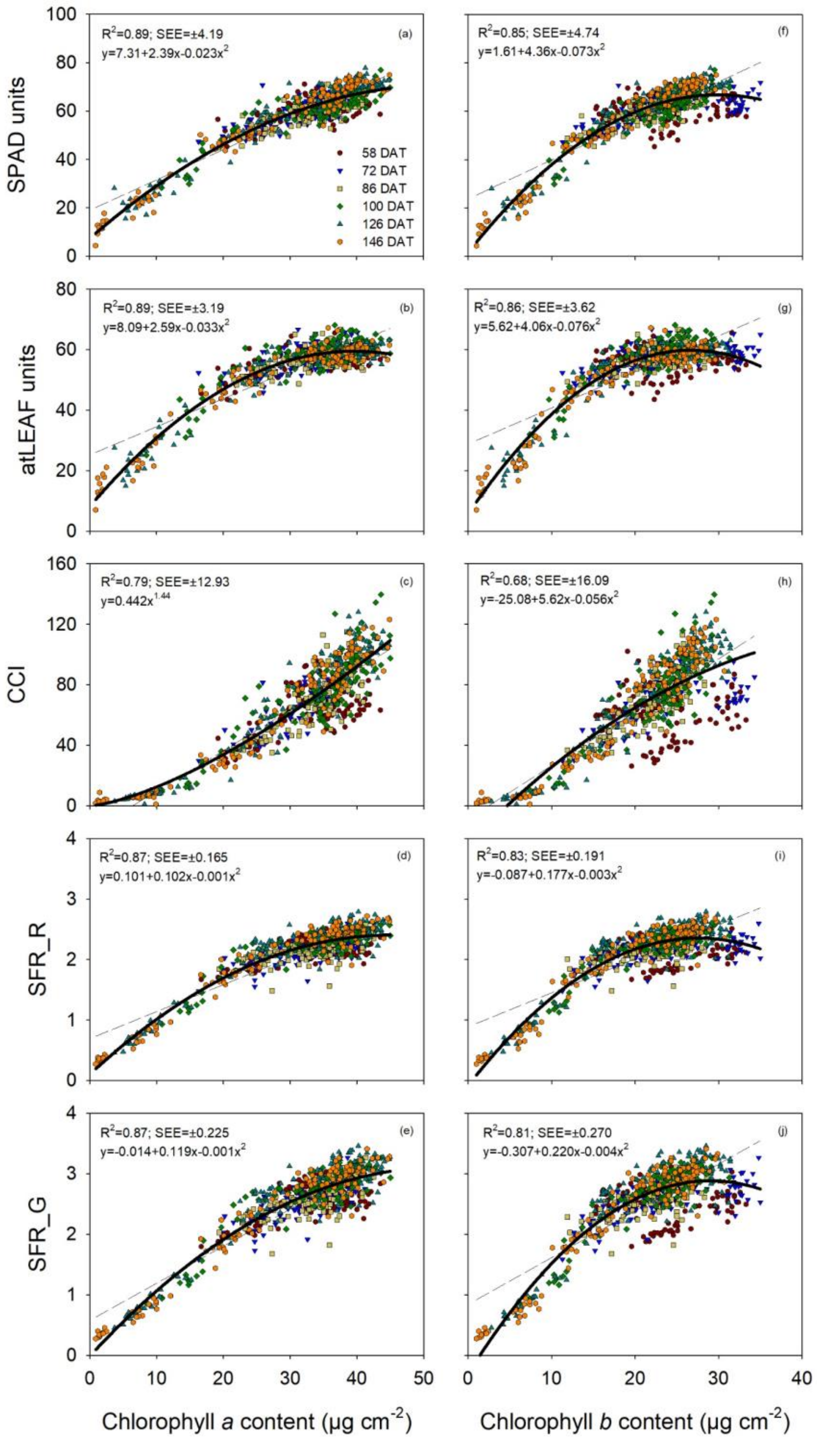

Figure S4. Relationships between chlorophyll *a* and chlorophyll *b* contents (µg cm^{-2}) and different chlorophyll meter measurements. Coefficient of determination (R^2), standard error of the estimate (±SEE) and equation of the regression are shown (solid lines). CCI is chlorophyll content index, measured with the MC-100 meter; SFR is Simple Fluorescence Ratio, either under red (SFR_R) or green (SFR_G) excitation, measured with the Multiplex sensor. Dotted lines represent the linear regression.
