# Supplementary material for: Different Responses of Various Chlorophyll Meters to Increasing Nitrogen Supply in Sweet Pepper
Source: Front Plant Sci. 2018 Nov 27;9:1752. doi: 10.3389/fpls.2018.01752 (PMC6277906; doi:10.3389/fpls.2018.01752)
Supplement: Figure S5 — Relationship between leaf N content (mg g-1) and chlorophyll a and chlorophyll b contents (μg cm-2). Coefficient of determination (R2), standard error of the estimate ( ± SEE) and equation of the regression are shown (solid lines). Dotted lines represent the linear regression. [file Image_5.pdf]

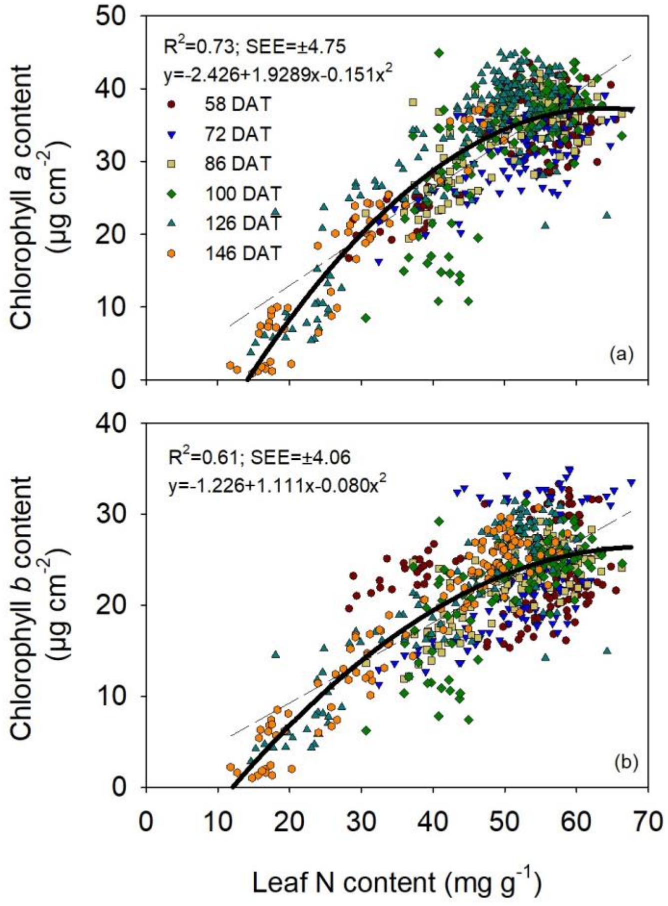

Figure S5. Relationship between leaf N content (mg g^{-1}) and chlorophyll *a* and chlorophyll *b* contents (µg cm^{-2}). Coefficient of determination (R^2), standard error of the estimate (±SEE) and equation of the regression are shown (solid lines). Dotted lines represent the linear regression.
