# Supplementary material for: Different Responses of Various Chlorophyll Meters to Increasing Nitrogen Supply in Sweet Pepper
Source: Front Plant Sci. 2018 Nov 27;9:1752. doi: 10.3389/fpls.2018.01752 (PMC6277906; doi:10.3389/fpls.2018.01752)
Supplement: Figure S6 — Relationship between measurements of different chlorophyll meters with the Simple Fluorescence Ratio under red excitation (SFR_R), measured with the Multiplex sensor. Coefficient of determination (R2) of the regression is shown; equations and standard errors of the estimate ( ± SEE) are in Supplementary Table S2. CCI is chlorophyll content index, measured with the MC-100 meter. [file Image_6.pdf]

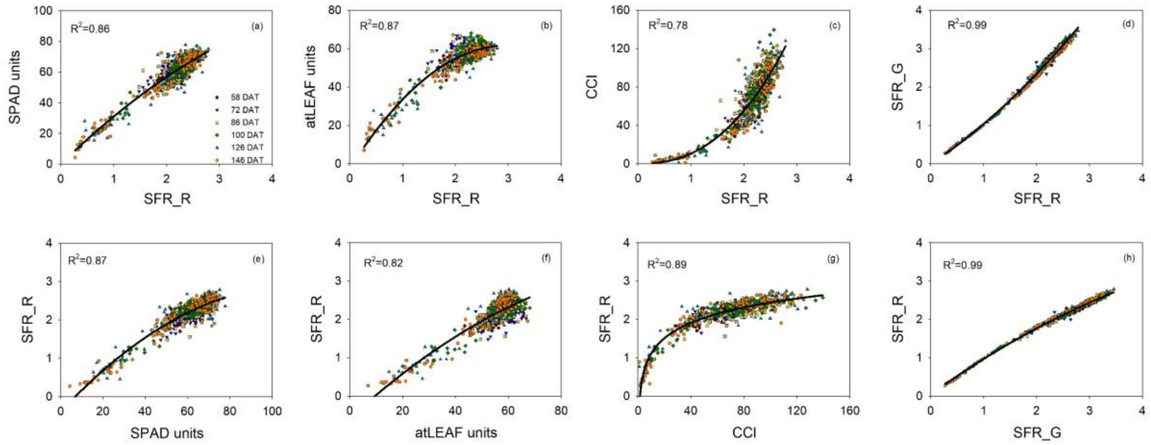

Figure S6. Relationship between measurements of different chlorophyll meters with the Simple Fluorescence Ratio under red excitation (SFR_R), measured with the Multiplex sensor. Coefficient of determination (R^2) of the regression is shown; equations and standard errors of the estimate (±SEE) are in Supplementary Table S2. CCI is chlorophyll content index, measured with the MC-100 meter.
