# Supplementary material for: Different Responses of Various Chlorophyll Meters to Increasing Nitrogen Supply in Sweet Pepper
Source: Front Plant Sci. 2018 Nov 27;9:1752. doi: 10.3389/fpls.2018.01752 (PMC6277906; doi:10.3389/fpls.2018.01752)
Supplement: Figure S7 — Comparison of relationships between: SPAD units and CCI values (a), CCI values and SPAD units (b), SPAD units and atLEAF units (c), and atLEAF units and SPAD units (d), of the present study and the literature. CCI is chlorophyll content index, measured with the MC-100 meter. [file Image_7.pdf]

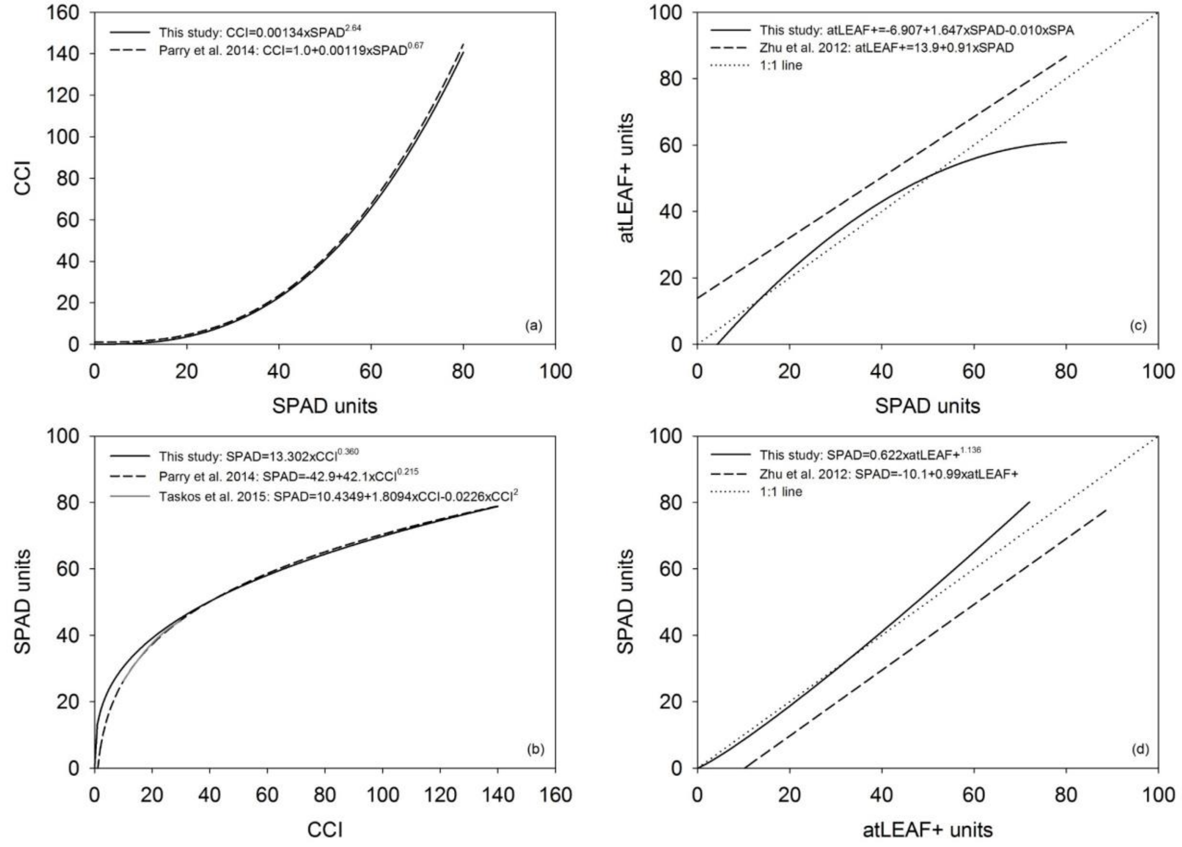

Figure S7. Comparison of relationships between: SPAD units and CCI values (a), CCI values and SPAD units (b), SPAD units and atLEAF units (c), and atLEAF units and SPAD units (d), of the present study and the literature. CCI is chlorophyll content index, measured with the MC-100 meter.
