# Supplementary material for: Different Responses of Various Chlorophyll Meters to Increasing Nitrogen Supply in Sweet Pepper
Source: Front Plant Sci. 2018 Nov 27;9:1752. doi: 10.3389/fpls.2018.01752 (PMC6277906; doi:10.3389/fpls.2018.01752)
Supplement: Table S1 — Equations to estimate chlorophyll a and chlorophyll b contents (μg cm-2) from measurements with different chlorophyll meters. Coefficient of determination (R2), standard error of the estimate ( ± SEE) and sample size (n) of the regression are shown. CCI is chlorophyll content index, measured with the MC-100 meter; SFR is Simple Fluorescence Ratio, either under red (SFR_R) or green (SFR_G) excitation, measured with the Multiplex sensor. [file Table_1.pdf]

Table S1. Equations to estimate chlorophyll *a* and chlorophyll *b* contents (µg cm^{-2}) from measurements with different chlorophyll meters. Coefficient of determination (R^2), standard error of the estimate (±SEE) and sample size (*n*) of the regression are shown. CCI is chlorophyll content index, measured with the MC-100 meter; SFR is Simple Fluorescence Ratio, either under red (SFR_R) or green (SFR_G) excitation, measured with the Multiplex sensor.

Chlorophyll	Chlorophyll meter	Equation	Regression	R^2	±SEE	*n*
Chlorophyll *a*	SPAD-502	Chl = -7.950 + 0.677 x SPAD	Linear	0.85	3.56	713
	atLEAF+	Chl = -5.774 + 0.430 x atLEAF + 0.0045 x $atLEAF^2$	Quadratic	0.77	4.42	719
	MC-100	Chl = 3.970 + 0.628 x CCI – 0.003 x CCI^2	Quadratic	0.88	3.33	667
	Multiplex	Chl = -5.929 + 17.872 x SFR_R	Linear	0.80	4.12	720
	Multiplex	Chl = -4.389 + 16.063 x SFR_G – 0.649 x SFR_G^2	Quadratic	0.83	3.79	720
Chlorophyll *b*	SPAD-502	Chl = 0.010 x $SPAD^{1.319}$	Power	0.73	3.42	713
	atLEAF+	Chl = 0.040 x $atLEAF^{1.57}$	Power	0.64	3.90	719
	MC-100	Chl = 3.989 + 0.398 x CCI – 0.002 x CCI^2	Quadratic	0.74	3.38	667
	Multiplex	Chl = -2.428 + 11.478 x SFR_R	Linear	0.65	3.90	720
	Multiplex	Chl = -2.035 + 11.013 x SFR_R – 0.589 x SFR_R^2	Quadratic	0.67	3.74	720
