# Supplementary material for: Different Responses of Various Chlorophyll Meters to Increasing Nitrogen Supply in Sweet Pepper
Source: Front Plant Sci. 2018 Nov 27;9:1752. doi: 10.3389/fpls.2018.01752 (PMC6277906; doi:10.3389/fpls.2018.01752)
Supplement: Table S2 — Equations to convert measurement units of different chlorophyll meters with the Simple Fluorescence Ratio under red excitation (SFR_R), measured with the Multiplex sensor. Coefficient of determination (R2), standard error of the estimate ( ± SEE) and sample size (n) of the regression are shown. CCI is chlorophyll content index, measured with the MC-100 meter; SFR_G is the Simple Fluorescence Ratio under green excitation. [file Table_2.pdf]

Table S2. Equations to convert measurement units of different chlorophyll meters with the Simple Fluorescence Ratio under red excitation (SFR_R), measured with the Multiplex sensor. Coefficient of determination (R^2), standard error of the estimate (±SEE) and sample size (*n*) of the regression are shown. CCI is chlorophyll content index, measured with the MC-100 meter; SFR_G is the Simple Fluorescence Ratio under green excitation.

Chlorophyll meter	Equation	Regression	R^2	±SEE	*n*
SPAD-502	$SPAD = -0.105 + 33.640 \times SFR\_R - 2.596 \times SFR\_R^2$	Quadratic	0.86	4.67	713
atLEAF+	$atLEAF = -2.607 + 43.343 \times SFR\_R - 7.320 \times SFR\_R^2$	Quadratic	0.87	3.56	719
MC-100	$CCI = 10.556 \times SFR\_R^{2.390}$	Power	0.78	13.27	667
Multiplex	$SFR\_G = -0.0087 + 0.910 \times SFR\_R + 0.131 \times SFR\_R^2$	Quadratic	0.99	0.05	720
Multiplex	$SFR\_R = -0.396 + 0.059 \times SPAD - 0.000265 \times SPAD^2$	Quadratic	0.87	0.16	713
	$SFR\_R = -0.577 + 0.063 \times atLEAF - 0.000243 \times atLEAF^2$	Quadratic	0.82	0.19	719
	$SFR\_R = -0.216 + 0.576 \times \ln(CCI)$	Logarithm	0.89	0.16	667
	$SFR\_R = 0.047 + 0.984 \times SFR\_G - 0.062 \times SFR\_G^2$	Quadratic	0.99	0.04	720
